# The Diagnosis and Management of Infraoccluded Deciduous Molars: A Systematic Review

**DOI:** 10.3390/children11111375

**Published:** 2024-11-12

**Authors:** Gianna Dipalma, Alessio Danilo Inchingolo, Lucia Memè, Lucia Casamassima, Claudio Carone, Giuseppina Malcangi, Francesco Inchingolo, Andrea Palermo, Angelo Michele Inchingolo

**Affiliations:** 1 Department of Interdisciplinary Medicine, University of Bari “Aldo Moro”, 70124 Bari, Italy; gianna.dipalma@uniba.it (G.D.); or a.inchingolo1@studenti.uniba.it (A.D.I.); or lucia.casamassima@uniba.it (L.C.); or claudio.mcarone@gmail.com (C.C.); or f.inchingolo@icloud.com (F.I.); or a.inchingolo3@studenti.uniba.it (A.M.I.); 2 Department of Specialized Clinical Sciences and Dentistry, Polytechnic University of Marche, 60121 Ancona, Italy; l.meme@staff.univpm.it; 3 College of Medicine and Dentistry, Birmingham B4 6BN, UK; andrea.palermo2004@libero.it

**Keywords:** infraocclusion, infraoccluded primary teeth, deciduous molar, dental anomalies

## Abstract

The infraocclusion (IO) of primary molars, often seen in retained deciduous teeth, is a common condition that presents significant challenges for pediatric oral health. It occurs when primary molars are positioned below the occlusal plane due to the absence of permanent successors, leading to complications such as misaligned teeth, impaired chewing, and long-term dental health issues. Objectives: This study examines IO prevalence, diagnosis, and treatment approaches. Methods: A systematic review following PRISMA guidelines was conducted, searching PubMed, Web of Science, and Scopus for articles from the last 15 years. Nine articles were included for qualitative analysis. Results: IO was associated with several complications, including root resorption, altered eruption of adjacent teeth, and space loss within the dental arch. Clinical and radiographic evaluations are key to early detection. Severe cases often require invasive treatments, such as tooth extraction and space maintenance, while mild cases could be monitored. Conclusions: IO is prevalent in pediatric dentistry and can lead to significant dental issues if untreated. Early detection and intervention are crucial for preventing complications like tooth misalignment and impacted premolars. Tailored treatment strategies based on severity, along with increased awareness among dental practitioners, are essential to improve long-term outcomes for affected children.

## 1. Introduction

**Figure 1:**
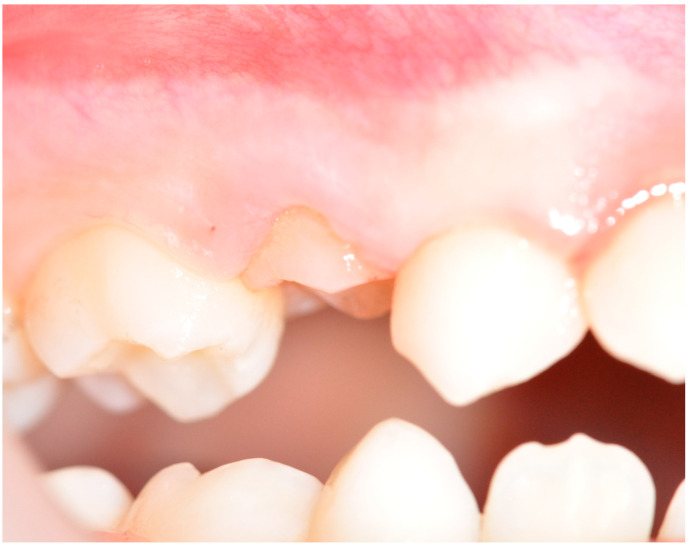
Clinical examination where there is IODM in a child of nine years old.

The IO (infraocclusion) of primary molars, particularly in the context of retained deciduous teeth, is a widespread dental condition that can present significant clinical challenges and implications for oral health [1,2,3,4,5]. It refers to the phenomenon where primary molars are positioned below the occlusal plane due to the absence of their permanent successors (Figure 1). This condition can lead to a cascade of complications, including misalignment of teeth, impaired chewing function, and potential long-term effects on overall dental health and development [6,7,8,9,10].

IO typically occurs when there is a disruption in the normal eruption sequence of permanent teeth. This disruption can be attributed to various factors, such as genetic predispositions, developmental anomalies, or environmental influences that hinder the timely eruption of the successor teeth [11,12,13,14,15]. The presence of IODMs (infraoccluded primary molars) is often accompanied by a range of associated dental complications, including a root resorption of the primary molars, alterations in the eruption patterns of neighbouring teeth, and the potential loss of space within the dental arch [16,17,18,19,20]. These issues not only affect the immediate dental alignment but may also have broader implications for facial aesthetics and function, impacting the child’s ability to chew and speak effectively [21,22,23,24,25].

To effectively address this condition, understanding how to diagnose it is crucial [26,27,28,29,30]. IO, which is defined as a tooth’s inability to stay aligned within the dental arch, has definite clinical symptoms that help with diagnosis and treatment. The apparent “submersion” of IO teeth to neighbouring teeth, which frequently causes a vertical disparity in the occlusal plane, is one of their primary clinical indications [30]. When nearby teeth move in the direction of the infraoccluded molar, it might cause space loss and affect the alignment of the entire arch. A lack of tooth movement and a characteristic sound when percussioned—often characterized as a “sharp” or “metallic” tone—are further consequences of IO teeth’s frequent ankylosis to the surrounding bone. The eruption of successor teeth may be delayed or prevented by this disorder, which can also interfere with normal exfoliation patterns [31,32].

**Figure 2:**
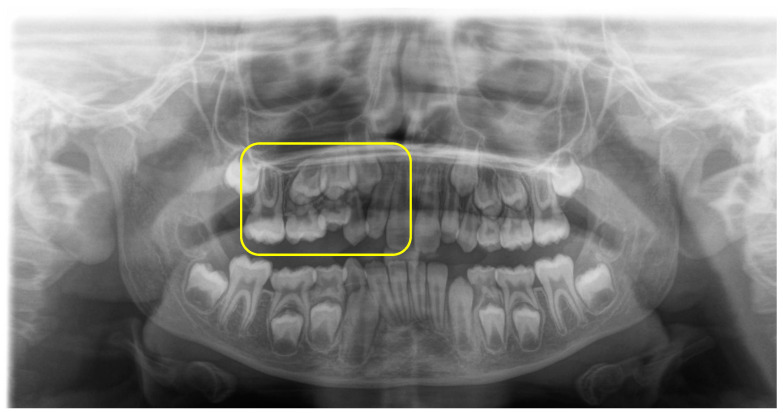
Orthopantomography (OPT)to determine the permanent successors’ status and the main molars’ position.

Diagnosis generally involves a combination of clinical examination and radiographic evaluation to assess the positioning of the primary molars and the status of their permanent successors (Figure 2). Dentists often look for specific signs such as the occlusal height of the molars, the presence of any root resorption, and the eruption status of adjacent teeth [33,34,35,36,37]. Identifying IO early is essential for preventing further complications, including the tilting of adjacent teeth and the potential impaction of successor premolars, which could complicate future orthodontic treatment [31,38,39,40,41].

The prevalence of IO highlights its clinical significance, as this condition is commonly observed in pediatric populations [42,43,44,45,46]. Early detection and intervention can help mitigate its effects, promoting healthier dental development and reducing the need for more invasive treatments later [47,48,49,50,51]. Given the importance of this topic, there is a pressing need for dental practitioners to enhance their awareness and understanding of IO [32,52,53,54,55].

This article aims to provide a comprehensive overview of IODMs, focusing on the condition’s prevalence, implications, and treatment strategies [56,57,58,59,60]. By synthesizing current knowledge and insights from recent research, we hope to illuminate the complexities of IO and foster better management approaches for this challenging dental anomaly in pediatric dentistry [61,62,63,64,65]. Ultimately, improving awareness and understanding of this condition among dental professionals can lead to better outcomes for affected children, ensuring a more positive trajectory for their oral health and overall well-being [66,67,68,69,70].

## 2. Materials and Methods

### 2.1. Search Processing

The current systematic review followed the PRISMA and International Prospective Register of Systematic Review Registry procedures (full ID: 598017). The following databases, PubMed, Web of Science, and Scopus, were examined from 1 September 2024 to 10 September 2024 to search for articles from the last 15 years (Table 1). The search strategy was created by combining terms relevant to the study’s purpose. The following Boolean keywords were applied: (((“molar infraocclusion”) OR (“primary molar infraocclusion”)) OR (infra-occluded molar)) OR (infraocclusion of primary molars).

### 2.2. Effect Measures

This review assessed the follow factors:

- Prevalence: Percentage of infraoccluded primary molars in study populations.
- Severity: Depth of infraocclusion classified as mild, moderate, or severe.
- Dental Anomalies: Odds ratios (ORs) for associations with conditions like agenesis and tipping.
- Occlusal Impact: Measurements of adjacent tooth tipping and space loss.
- Treatment Outcomes: Success rates of interventions, such as spontaneous eruption.

These measures provided a detailed understanding of infraocclusion’s clinical impact across studies.

### 2.3. Inclusion and Exclusion Criteria

The reviewers worked in groups to assess all relevant studies that analyzed or compared the prevalence and/or the incidence of IODMs, according to the following inclusion criteria:

- Studies with open access written in English;
- Studies that performed the research “in vivo” or in “humans”;
- Case–control studies, cohort studies, RCTs;
- Studies that were published in the last 15 years;
- Only IODMs.

Studies that fulfil at least one of the following exclusion criteria were excluded: reviews, case reports, and series; letters to the authors; animal models; and in vitro studies.

The evaluation of study eligibility was performed in several steps:

- Screening: Studies that did not fit the fundamental inclusion criteria were first weeded out of the titles and abstracts.
- Full-Text Review: Following that, two impartial reviewers looked over the entire text to make sure it met all inclusion and exclusion requirements.
- Resolution of Discrepancies: Any disputes were settled by dialogue or the advice of a third reviewer.

### 2.4. PICo Question

The PICo question addressed was “What are the management and/or treatment options for IODMs in patients with deciduous, mixed, or permanent dentition in children and adults?”

I. Population (P):

Patients with deciduous, mixed, or permanent dentition.

II. Phenomenon of Interest (I):

Management and/or treatment of IODMs.

III. Context (Co):

In children and adults.

### 2.5. Data Processing

Four independent reviewers (A.D.I., L.C., C.C., and G.M.) assessed the quality of the included studies using specified criteria such as selection criteria, methods of outcome evaluation, and data analysis. This enhanced “risk of bias” tool additionally includes quality standards for selection, performance, detection, reporting, and other biases. All differences were settled through conversation or collaboration with other researchers (G.D., A.L., A.P., and A.M.I). The reviewers screened the records according to the inclusion and exclusion criteria. Doubts have been resolved by consulting the senior reviewer (F.I.). The selected articles were downloaded into Zotero 6.0.36.

## 3. Results

### 3.1. Characteristics of Included Articles

**Figure 3:**
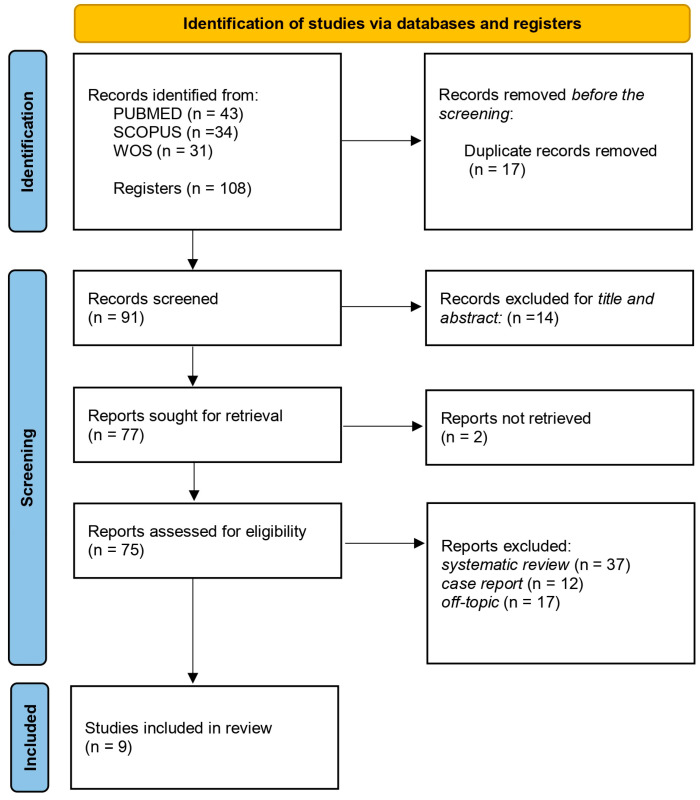
Literature search PRISMA flow diagram and database search indicators.

Figure 3 shows the flow diagram of a systematic review carried out using the Preferred Reporting Items for Systematic Reviews and Meta-Analyses (PRISMA) reporting criteria.

The diagram describes the search strategy, inclusion, and exclusion of publications at each stage of detection. A total of 108 publications were identified in three databases, including PubMed (43), Web of Science (31), and Scopus (34), after the duplicates were deleted (17). The analysis of titles and abstracts led to the exclusion of 14 articles for being unrelated to the topic. The remaining seventy-seven records were read, deleting two of them that were not retrieved. Then, 66 articles did not fill the inclusion criteria. The evaluation includes a total of nine publications for qualitative analysis.

### 3.2. Descriptive Summary of Item Selection

The Table 2 shows Descriptive Summary of Item Selection.

### 3.3. Quality Assessment and Risk of Bias of Included Articles

The risk of bias was measured with the “Risk of bias in non-randomized studies-of intervention” or ROBINS I-tool and included the nine studies reported in Table 3. Regarding bias due to confounding, all of them have some concerns (D1). The bias resulting from measurement is a parameter with a low risk of bias for all studies (D2). Only four studies have a low risk of bias due to participants while the others have some concerns (D3). There is no information about post-exposure bias in five studies, while three have a low risk of bias (D4). Bias due to missing data is low in five studies (D5). The selection bias of the reported results is low in two of the studies, and, in seven of the studies, there are some concerns (D6). The results show that four studies have a low risk of bias, and five have a medium risk of bias.

### 3.4. Implications for Practice, Policy, and Research

- Practice: Routine screening for infraoccluded molars in children’s dental exams is essential for early intervention and preventing complications like tooth tipping and space loss.
- Policy: Standardized diagnostic and intervention guidelines are needed to improve detection and management across different regions.
- Research: Future studies should focus on the long-term outcomes of IO treatments, the genetic factors involved, and developing consistent diagnostic criteria to guide treatment.

## 4. Discussion

### 4.1. IO and Causes

There are several causes of infraocclusion (IO) of deciduous molars; however, they mostly fall into three groups:

- environmental
- developmental
- hereditary

Hereditary influences are one type of genetic component; those who have a family history of infraocclusion or related abnormalities (such as tooth agenesis or hypodontia) are more likely to develop IO [45]. Developmental factors can prevent the molar from erupting and keep it at a lower level than neighbouring teeth by interfering with the normal eruption sequence, which is frequently connected to anomalies in the cementum or periodontal ligament [75]. Local impacts including trauma, early root resorption, or ankylosis—a condition in which the union of the tooth root and alveolar bone limits vertical movement—can be considered environmental variables [31]. Together, these factors can lead to IO by disrupting the natural balance and eruption mechanisms in the dentition, resulting in varied clinical outcomes based on the severity and combination of influencing factors.

### 4.2. IO Diagnosis Techniques

Several imaging modalities offer differing degrees of specificity, precision, and usefulness when assessing infraocclusion (IO).

The most often utilized methods are as follows:

- Panoramic Radiographs: This is a widely accessible, quick, and cost-effective option for identifying infraocclusion, especially in initial assessments. However, panoramic radiographs offer limited resolution and can distort tooth size and position, which may hinder precise measurements of the infraocclusion depth or subtle structural changes [80].
- Periapical Radiographs: These provide greater detail for IO teeth in specific areas, allowing for clearer visualization of root resorption, periodontal ligament (PDL) status, and bone structure around the infraoccluded tooth. While useful for localized diagnostics, periapical radiographs lack the full-arch perspective that panoramic radiographs offer, which can be limiting for treatment planning [26].
- Cone-Beam Computed Tomography (CBCT): CBCT provides a 3D view, offering highly detailed visualization of the tooth structure, root morphology, surrounding bone, and PDL space, which is ideal for evaluating ankylosis and precise measurements of IO depth. Despite its accuracy, CBCT is often more costly and has a higher radiation dose than 2D techniques, so it is generally reserved for complex cases or when conventional radiographs are inconclusive [17].
- Intraoral Photography: Though not technically a radiographic technique, intraoral photography allows clinicians to document the occlusal relationship visually, providing an additional reference point for infraocclusion’s impact on aesthetics and functionality. This method, however, cannot visualize underlying bone or root conditions and is generally used as a supplementary tool alongside radiographic technique [71].

Each technique has its strengths: panoramic and periapical radiographs are suitable for initial assessments and monitoring, whereas CBCT is best for detailed diagnostics and treatment planning in severe or complex IO cases. Selecting the appropriate imaging method depends on the clinical presentation, the suspected severity of IO, and the need for precise anatomical details.

### 4.3. IO Prevalence

The infraocclusion of primary molars occurs in approximately 4–8% of children, though this rate varies by age, population, and diagnostic criteria. For example, studies have shown prevalence rates of around 4.3% among children aged 3–15 years, with IO typically presenting between the ages of 6 and 9 [78].

Prevalence can differ across populations. For instance, a study in Valdivia, Chile found that 41.78% of 7- and 8-year-old children showed some degree of infraocclusion in primary molars. Another study among Arabian children found IO in 7.4% of primary molars, with a higher frequency among boys [74,75].

Within populations, most infraoccluded molars are mild, with severe cases (more than 5 mm of submersion) constituting about 1.5–2.6% of affected teeth. For example, studies often report that over 80% of infraoccluded cases are mild, with a smaller proportion (up to 18%) being severe [38].

### 4.4. IO and Root Resorption

The study by Hvaring C. L. et al. evaluated IO, root resorption, and restorations in retained primary mandibular molars without permanent successors, using a sample of 188 molars from 111 patients (mean age 12.6 years) [71]. Clinically significant IO was found in 43.6% of cases, with 18.8% classified as severe. Mesial and distal root resorption showed significant variation (*p* = 0.01). Most molars (78.4%) had no restorations, and a correlation was found between root resorption and IO. Age was weak but significantly associated with both factors, while gender showed no effect. IO was deemed the most critical factor for prognosis [81,82,83,84,85].

### 4.5. Severe IO and Associated Anomalies

Shalish M. et al. aimed to assess the clinical features and treatment of severe IO, and its relationship with other dental anomalies. The sample consisted of 25 orthodontic patients, aged 7–14 years (mean 10.1 years), with at least one deciduous molar submerged over 5 mm (meaning 9 mm). Adjacent teeth were severely tilted, space was lost, and all successor premolars were impacted. Treatment involved regaining space, surgically extracting the IODM and maintaining space, leading to spontaneous eruption of impacted premolars in 95% of cases. A significant increase in other dental anomalies (e.g., tooth AG, displaced canines) was observed, suggesting a shared genetic cause. Early markers for deep submersion include severe tooth tilting and space loss [72,86,87,88,89].

Diaz Schiappacasse F. et al.’s objective was to determine the prevalence of IO in primary molars of 7- and 8-year-old children in Valdivia, Chile [74]. A cross-sectional descriptive study was conducted, evaluating 359 children in educational institutions in the region. The presence and severity of IO in primary molars were assessed using the Brearley and McKibben classification. To analyze statistically significant differences between sexes and the presence of IO, the chi-square test was applied, while analysis of variance (ANOVA) was used to evaluate the localization and severity of IO, with a significance level set at *p* < 0.05.

The results indicated that 41.78% of the evaluated children exhibited IO. In terms of severity, 82.06% of cases were classified as mild, 15.28% as moderate, and 2.66% as severe. No significant differences were found in the prevalence of IO between sexes. However, statistically significant differences were observed when evaluating the localization and degree of severity (*p* < 0.05).

In conclusion, this study reveals a high prevalence of IO among 7- and 8-year-old children in Valdivia, Chile, highlighting the need for increased clinical attention to this condition in the pediatric population [74,90,91,92,93].

Alshaya S. I. et al. analyzed the prevalence and characteristics of IO among Arabian children in primary dentition and its associated dental anomalies [75]. A retrospective analysis was conducted on digital OPT from 542 children who attended the pediatric dental clinic at Majmaah University, Saudi Arabia, from January 2019 to May 2021. IO was identified in 40 children, affecting a total of 65 primary molars, predominantly in males (90%) and mainly in the mandibular arch (n = 48).

Unilateral IO was more common than bilateral (62.5% vs. 37.5%), with single molars affected in 50% of cases. The mandibular second primary molar was the most frequently involved, while the maxillary first primary molar was less affected. The majority of IOs were mild (75%), with moderate (23.5%) and severe (1.5%) cases also noted. Additionally, IO was frequently associated with dental anomalies such as hypodontia (12.5%) and supernumerary teeth (5%) [94,95,96,97,98].

IO is commonly observed in the mandibular second primary molars among Arabian children, with unilateral cases generally being mild. The presence of numerical anomalies like hypodontia and supernumerary teeth is also associated with IO in this population [75,99,100,101,102].

### 4.6. Dental Variations and Developmental Impact

The research by Odeh R. et al. investigated the prevalence of specific dental variations associated with IO and assessed its impact on dental development and tooth size in singletons and twins [73]. Two samples were analyzed: the first consisted of 1.454 OPT from singleton children aged 8 to 11 years, and the second included dental models of 202 pairs of monozygotic and dizygotic twins within the same age range. IO was quantified using Adobe Photoshop CS5 to measure the extent in millimetres from the radiographs and 2D images of the dental models [73].

The findings revealed a significant association between IO and altered canine eruption positions, as well as the lateral incisor complex, including AG and reduced tooth size (*p* < 0.001). However, no significant correlation was found between IO and the AG of premolars. Moreover, dental age assessments indicated that individuals with IO exhibited delayed dental development compared to controls, with primary mandibular canines being significantly smaller in size in the IO group (*p* < 0.05) [73].

These results suggest a pleiotropic effect, indicating that the presence of various dental anomalies, delayed development, and reduced tooth size may stem from shared underlying genetic and/or epigenetic factors [73,103].

Dental ankylosis is a serious condition defined as the fusion of dentin or cementum with the alveolar bone, leading to the progressive replacement of the periodontal ligament with bone tissue [80,104,105,106,107]. The study by Esian D. et al. determined the prevalence, location, severity, and associations of dental ankylosis in primary molars and its relationship with other dental anomalies, such as the AG of permanent teeth [76]. A total of 150 panoramic X-rays were analyzed from patients aged six to twelve years at a dental clinic and the Paediatric Dentistry Department of UMFST in Targu-Mures, Romania.

The results showed a higher prevalence of ankylosis (72%) in children aged six to nine years compared to 28% in those aged ten to twelve. There was no association between ankylosis and gender, but most cases were found in the lower arch, particularly in quadrant three, with the first primary molar being the most affected [76]. Mild to moderate IO was observed, resulting in a minimal functional impact on the dental arch and neighbouring teeth. The differences from previous studies, especially regarding localization, may stem from sample size and diagnostic methods [76,108,109,110]. Dental ankylosis occurs frequently in early mixed dentition, predominantly affecting the lower arch. Early diagnosis through signs such as IO and lack of dental mobility is crucial to prevent further complications [76,111,112,113,114,115].

Mandibular second premolar (M2P) agenesis can lead to several dental complications, including the retention of the second primary molar (2pm), IO, and alterations in alveolar structure, as well as the supra-eruption of opposing teeth and movement of adjacent teeth [116,117,118,119,120]. Calheiros-Lobo M. J. et al.’s study evaluated the lifespan of the 2pm as a substitute tooth in cases of M2P agenesis, particularly focusing on root quality and occlusal adaptation within a low-income population. A total of 12.949 OPT were examined, involving 61 patients (25 males and 36 females, aged 7–36 years), categorized based on whether the first permanent molar (FPM) and second permanent molar (SPM) were in occlusion [77].

The results revealed that while the study design was cross-sectional, there were notable correlations between age and factors such as root resorption, IO, and the distance between the FPM and primary molar. The degree of 2pm root resorption was found to increase with age, especially when the SPM was also in occlusion [121,122]. The mesial movement of adjacent teeth was absent across all groups. Although the 2pm frequently maintained occlusion, IO increased with age, particularly during critical periods of 11–15 years and 21–25 years, which were identified as significant for primary tooth loss.

In conclusion, the 2pm can remain functional in the mandibular arch for up to 25 years. A conservative, no-intervention approach supported by clinical and radiographic evaluations should be considered in cases devoid of orthodontic issues or financial limitations.

Akgol B. B. et al. investigated the prevalence, classification, accompanying findings, and treatment modalities related to IODMs, aiming to categorize these molars based on the severity of IO and evaluate the corresponding treatment approaches. The research included a sample of 3132 subjects aged 3 to 15 years, revealing an overall prevalence of 4.3% for IO, with the condition typically manifesting between the ages of 6 and 9, primarily affecting mandibular primary molars [78].

Treatment strategies varied according to the severity of IO, with more invasive procedures required for severe cases [123,124,125,126,127]. The accompanying findings highlighted adjacent teeth tipping, significant midline deviation towards the affected side, and an increased prevalence of dental caries. Notably, AG of succeeding premolars was observed in 2% of IODM, with higher extraction rates noted when the successor tooth was positioned mesially or distally.

Overall, the findings provide dental practitioners with valuable insights into the severity and distribution of treatment interventions for IO. The study emphasizes the necessity for timely and personalized therapeutic strategies, particularly for more severe cases, to enhance patient outcomes [128,129,130,131,132].

Marcianes M. et al. examined the potential associations between MIH, a developmental enamel defect with uncertain etiology, and two specific components of the Dental Anomaly Pattern (DAP): AG and IODMs [79]. Given that the DAP encompasses various morphological, numerical, and eruptive anomalies that often occur together, establishing a genetic link between MIH and these anomalies could strengthen the hypothesis of a shared etiology.

The research analyzed standardized intraoral photographs and OPT of 574 children aged 8 to 14 years, comprising 287 with MIH and 287 without. The study compared the frequencies of AG and IODM in both groups. The results indicated that the frequencies of AG were 7% in the MIH group and 8% in the non-MIH group (*p* = 0.751), while the frequencies of IODM were 27% and 19.2%, respectively (*p* = 0.082). These findings suggest that children with MIH do not have a higher prevalence of AG or IODM compared to their counterparts without MIH.

Consequently, the results do not support the inclusion of MIH in the DAP framework. However, the authors highlight the need for further investigations to comprehensively explore any possible associations between these dental anomalies [133,134,135,136,137].

### 4.7. IO Management

The severity of the problem, the age of the patient, and the existence of permanent successors all influence how infraoccluded teeth are now managed:

- For mild cases without progressive infraocclusion or complications, periodic observation is often sufficient, especially if successor teeth are expected to erupt normally;
- When IO teeth affect occlusion, restorations like composite resin build-ups can raise the tooth’s occlusal height to align with the dental arch, preserving space and function temporarily;
- In severe cases, or when significant tipping or space loss occurs, extraction followed by space maintenance (e.g., a space maintainer) is recommended. This approach prevents adjacent teeth from shifting into the gap, allowing space for future dental implants or orthodontic treatment;
- For cases involving significant space loss, orthodontic appliances may be used to regain lost space or to guide the alignment of impacted permanent teeth;
- In adults or in cases where there is no permanent successor, implants or prosthetic replacements can be considered after extraction, particularly to restore function and esthetics in the long term.

The treatment plan is customized for each patient, with attention to maintaining arch stability and reducing interference with overall dental growth.

## 5. Conclusions

In summary, the study of IO in primary molars reveals several key points regarding its prevalence, causes, diagnosis, and management. The following conclusions can be drawn:

- Prevalence: IO of primary molars is a common condition in children, requiring early diagnosis to prevent further dental complications.
- Causes and Complications: It results from disruptions in tooth eruption, leading to misalignment, root resorption, and space loss, affecting oral function and aesthetics.
- Diagnosis: Clinical and radiographic evaluations are essential to detect IO early and prevent complications like tooth tilting and impacted premolars.
- Treatment: Management depends on the severity, with mild cases monitored and severe ones requiring intervention like extraction or space maintenance.
- Genetic Factors: IO is linked to other dental anomalies, suggesting a genetic component that requires tailored treatment strategies.
- Long-term Impact: Untreated IO can cause delayed tooth eruption and increase the risk of caries, stressing the importance of timely treatment.

## 6. Limitations of the Study and Future Directions

Several issues restrict the evidence in this review, including variances in the diagnostic criteria and severity categorization for infraocclusion, sample variability across age groups and countries that may impact comparability, and variations in study design (case–control, cohort, and cross-sectional). Smaller sample numbers in some studies may limit generalizability, and the absence of longitudinal research restricts our understanding of the course and long-term consequences of infraoccluded molars. To have a better understanding of the prevalence, development, and treatment effects of infraocclusion, more comprehensive and systematic research is necessary.

For future research, longitudinal studies are essential to understand the natural progression and long-term effects of infraocclusion and to evaluate the outcomes of different treatment approaches. Standardizing diagnostic criteria and severity classifications would also enhance comparability across studies. Further investigation into genetic and environmental factors contributing to infraocclusion may offer insights into early detection and prevention, supporting a more targeted approach in clinical practice.

## Figures and Tables

**Table 1:** Indicators for database searches.

Article-screening strategy	KEYWORDS: “A”: molar IO; “B”: primary molar IO; “C”: infra-occluded molar; “D”: IO of primary molars.
	Boolean Indicators: “A” OR “B” OR “C” OR “D”.
	Timespan: 1 September 2024 to 10 September 2024
	Electronic databases: PubMed; Scopus; Web of Science.

**Table 2:** This table summarizes the relevant details from the article, including the authors, type of study, objectives, materials used, and the main findings (outcomes) [71,72,73,74,75,76,77,78,79].

Authors and Years	Type of Study	Aim of the Study	Materials	Outcomes
Hvaring, C.L. et al. (2014) [71]	Retrospective observational Study	To assess IO, root resorption, and restorations in retained primary mandibular molars without permanent successors in patients with severe hypodontia.	111 patients, 188 retained primary mandibular second molars, OPT, Facad software for measurements.	IO was found in 43.6% of patients, with severe cases in 18.8%. A significant correlation was observed between IO and root resorption. Age also showed a weak correlation with both IO and root resorption. Restorations were not significant in prognosis.
Shalish, M. et al. (2014) [72]	Retrospective Study	To evaluate treatment modalities for deep submersion and its association with other dental anomalies.	25 orthodontic patients with IODM, dental records, radiographs	Treatment resulted in spontaneous eruption in 95% of cases. Increased prevalence of dental anomalies in deep submersion cases.
Odeh, R. et al. (2015) [73]	Retrospective Study	To investigate the association between IO, dental variations, and dental development in singletons/twins	1454 radiographs of singletons (8–11 years), and 202 twins (8–11 years) dental models.	Significant association of IO with canine eruption anomalies and lateral incisor complex. Delayed dental development and smaller mandibular canines in IO cases.
Díaz Schiappacasse, F. et al. (2020) [74]	Cross-sectional study	To determine the prevalence of IO in primary molars of children aged 7 and 8 in Valdivia, Chile	Examination of 359 children in educational institutions, using the Brearley and McKibben classification for IO evaluation. Statistical analyses with chi-square and ANOVA tests	41.78% prevalence of IO. - 82.06% of cases were mild, 15.28% moderate, and 2.66% severe. - Statistically significant differences in IO location and severity. (*p* < 0.05)
Alshaya, S.I. (2022) [75]	Retrospective cross-sectional study	Analyze the prevalence, distribution, and characteristics of IO in primary dentition among Arabian children and its associated dental anomalies.	542 children aged 4–12 years from the pediatric dental clinic at Majmaah University, Saudi Arabia	IO is common in mandibular second primary molars, predominantly mild, and associated with anomalies like hypodontia. Regular follow-up is advised.
Eşian, D. et al. (2022) [76]	Retrospective cross-sectional radiographic analysis	To analyze the prevalence, characteristics, and associated dental anomalies of IO among Arabian children in primary dentition	OPT of 542 children attending the pediatric dental clinic at Majmaah University, Saudi Arabia, from January 2019 to May 2021.	7.38% prevalence of IODM. - IO was more common in males (90%) and mandibular second primary molars (58%). - Hypodontia (12.5%) and supernumerary teeth (5%) were the most frequently associated anomalies.
Calheiros-Lobo, M.J. et al. (2022) [77]	Retrospective cross-sectional	To evaluate the lifespan and functionality of retained second primary molars in cases of second premolar agenesis (AG), particularly the extent of IO and root resorption.	2.949 OPTs were analyzed from patients aged 7–36 years. A sample of 61 patients was selected for analysis based on retention of second primary molars.	Second primary molars remained functional for up to 25 years. - IO and root resorption increased with age, with critical loss phases at ages 11–15 and 21–25. - Mesial movement of adjacent teeth was absent. - A non-intervention approach could be considered in cases without other complications.
Akgöl, B.B. et al. (2024) [78]	Retrospective cross-sectional study	To investigate the prevalence, classification, accompanying findings, and treatment interventions related to IODMs in children.	4.828 OPT of children aged 3 to 15 years.	Prevalence of IO was 4.3%. - Most cases (84.8%) were classified as mild (Group I), with more severe cases requiring extraction. - Accompanying findings included tipping of adjacent teeth, midline shifts, and increased caries. - Premolar AG was identified in 2% of cases, and extraction was more frequent when the successor tooth was malpositioned.
Marcianes, M. et al. (2024) [79]	Observational cross-sectional study	To explore potential associations between molar–incisor hypomineralization (MIH) and two specific dental anomalies: AG and IODM	Sample of 574 children aged 8–14 years, 287 with MIH and 287 without MIH. OPT and standardized intraoral photographs were used.	- No significant association between MIH and dental AG (7% in MIH group vs. 8% in non-MIH group). - No significant association between MIH and IODM (27% vs. 19.2%, *p* = 0.082).

**Table 3:** Bias assessment.

Authors (Year)	D1	D2	D3	D4	D5	D6	Overall
Hvaring, C.L. et al. (2014) [71]	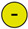	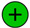	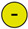	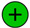	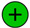	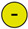	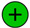
Shalish, M. et al. (2014) [72]	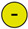	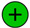	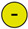	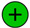	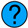	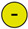	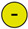
Odeh, R. et al. (2015) [73]	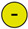	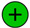	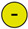	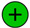	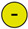	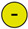	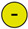
Díaz Schiappacasse, F. et al. (2020) [74]	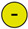	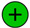	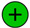	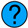	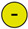	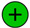	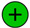
Alshaya, S.I. et al. (2022) [75]	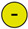	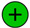	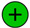	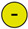	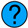	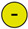	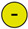
Eşian, D. et al. (2022) [76]	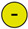	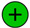	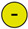	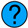	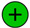	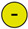	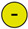
Calheiros-Lobo, M.J. et al. (2022) [77]	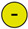	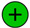	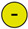	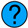	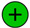	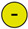	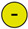
Akgöl, B.B. et al. (2024) [78]	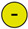	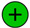	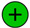	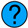	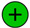	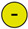	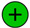
Marcianes, M. et al. (2024) [79]	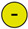	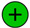	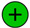	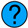	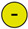	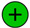	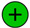
Domains:			Judgement:				
D1: Bias due to confounding.			Very High			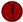	
D2: Bias arising from the measurement of the exposure.			High			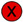	
D3: Bias in the selection of participants in the study (or in the analysis).			Some Concerns			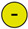	
D4: Bias due to post-exposure interventions.			Low			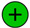	
D5: Bias due to missing data.			No Information			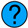	
D6: Bias arising from measurement of the outcome.							

## Data Availability

Data are contained within the article.

## Abbreviations

AG	Agenesis
DAP	Dental Anomaly Pattern
FPM	First permanent molar
IO	Infraocclusion
IODM	Infraoccluded deciduous/primary molars
M2P	Mandibular second premolar
MIH	Molar–incisor hypomineralization
OPT	Orthopantomography
2pm	Second primary molar
SPM	Second permanent molar
